# *Plasmodium falciparum* *pfhrp2* and *pfhrp3* Gene Deletions and Relatedness to Other Global Isolates, Djibouti, 2019–2020

**DOI:** 10.3201/eid2810.220695

**Published:** 2022-10

**Authors:** Eric Rogier, Jessica N. McCaffery, Mohamed Ali Mohamed, Camelia Herman, Doug Nace, Rachel Daniels, Naomi Lucchi, Sophie Jones, Ira Goldman, Michael Aidoo, Qin Cheng, Edie A. Kemenang, Venkatachalam Udhayakumar, Jane Cunningham

**Affiliations:** Centers for Disease Control and Prevention, Atlanta, Georgia, USA (E. Rogier, J.N. McCaffery, C. Herman, D. Nace, N. Lucchi, S. Jones, I. Goldman, M. Aidoo, V. Udhayakumar);; Oak Ridge Institute for Science and Education, Oak Ridge, Tennessee, USA (J.N. McCaffery);; Hôpital Général Peltier, Djibouti City, Djibouti (M.A. Mohamed);; Harvard T.H. Chan School of Public Health, Boston, Massachusetts, USA (R. Daniels);; Broad Institute, Cambridge, Massachusetts, USA (R. Daniels);; Australian Defence Force Malaria and Infectious Disease Institute, Brisbane, Queensland, Australia (Q. Cheng);; World Health Organization, Geneva, Switzerland (E.A. Kemenang, J. Cunningham)

**Keywords:** *Plasmodium falciparum*, Djibouti, pfhrp2, pfhrp3, parasite relatedness, malaria, parasites, vector-borne infections

## Abstract

Deletions of *pfhrp2* and paralogue *pfhrp3* (*pfhrp2/3*) genes threaten *Plasmodium falciparum* diagnosis by rapid diagnostic test. We examined 1,002 samples from suspected malaria patients in Djibouti City, Djibouti, to investigate *pfhrp2/3* deletions. We performed assays for *Plasmodium* antigen carriage, *pfhrp2/3* genotyping, and sequencing for 7 neutral microsatellites to assess relatedness. By PCR assay, 311 (31.0%) samples tested positive for *P. falciparum* infection, and 296 (95.2%) were successfully genotyped; 37 (12.5%) samples were *pfhrp2*+/*pfhrp3*+, 51 (17.2%) were *pfhrp2*+/*pfhrp3*–, 5 (1.7%) were *pfhrp2*–/*pfhrp3*+, and 203 (68.6%) were *pfhrp2*–/*pfhrp3*–. Histidine-rich protein 2/3 antigen concentrations were reduced with corresponding gene deletions. Djibouti *P. falciparum* is closely related to Ethiopia and Eritrea parasites (pairwise G_ST_ 0.68 [Ethiopia] and 0.77 [Eritrea]). *P. falciparum* with deletions in *pfhrp2/3* genes were highly prevalent in Djibouti City in 2019–2020; they appear to have arisen de novo within the Horn of Africa and have not been imported.

Diagnosis and appropriate case management of *Plasmodium falciparum* infection has greatly improved in many malaria-endemic settings through the use of rapid diagnostic tests (RDTs) that detect the histidine-rich protein 2 (HRP2) antigen (*1*). As the only *Plasmodium* species infecting humans to produce this antigen, the *P. falciparum* parasite expresses HRP2 in abundance and releases it into the bloodstream during blood-stage infection, making this marker a very sensitive and specific target for falciparum malaria (*1*,*2*). The *pfhrp2* gene is located on chromosome 8 of the parasite genome, and a paralogous gene (*pfhrp3*) is located on chromosome 13. The 2 protein products share common epitopes for diagnostic antibodies, enabling the HRP3 antigen to also be detected to some extent by HRP2-based RDTs (*3*–*6*).

*P. falciparum* produces large quantities of these antigens during human blood-stage infection, but their biologic functions are not well elucidated, and *pfhrp2*-deleted and *pfhrp3*-deleted parasites still complete the human–mosquito lifecycle successfully (*7*). Reports of these gene deletions have increased over the past decade from multiple countries in Africa, South America, and Asia (https://apps.who.int/malaria/maps/threats) (*8*). For countries that rely on HRP2-based RDTs for diagnosis of *P. falciparum* infection, those reports affirm the need to monitor the performance of this tool because deleted parasites could emerge and elicit false-negative results.

*P. falciparum* infection represents ≈99.7% of all malaria cases in sub-Saharan Africa, and ≈300 million HRP2-based RDTs are used in this region annually (*9*). Studies in the east Africa countries of Eritrea (*10*) and Ethiopia (*11*,*12*) have found high prevalence of *pfhrp2/pfhrp3* deletions, forcing changes away from HRP2-based RDTs to accurately diagnose *P. falciparum* infections in these countries. Furthermore, it is unknown whether these deleted genotypes are a result of importation and expansion or whether de novo deletions are arising from local *P. falciparum* lineages. A recent report from Djibouti, which borders both Eritrea and Ethiopia, investigated 79 *P. falciparum*–infected patients and found ≈80% of parasites were lacking both *pfhrp2* and *pfhrp3* genes (*13*). Triggered by health workers’ reports of false-negative RDT results, we report data from an investigation of 1,002 suspected malaria patients enrolled in Djibouti City during December 2019–March 2020. Data were generated for infection-causing *Plasmodium* species, *pfhrp2/3* genotype, concordance with laboratory antigen detection, and relatedness to other global *P. falciparum* parasites.

## Materials and Methods

### Patient Enrollment and Ethics Statement

This activity was considered by the Ministry of Health of Djibouti, the World Health Organization (WHO) Ethical Review Committee, and Centers for Disease Control and Prevention (CDC) human subjects office as nonresearch and as public health surveillance (0900f3eb81abbef6). In December 2020, a study was initiated to investigate presence of *pfhrp2/3* deletions in Djibouti because of 4 specimens that tested negative by HRP2-based RDT (CareStart Malaria Combo RDT; Access Bio, https://accessbio.net) but were positive for *P. falciparum* lactate dehydrogenase (pf-LDH) (Bioline Malaria Ag Pf [HRP2/pLDH] Test; Abbott, https://www.abbott.com) and also confirmed for *P. falciparum* infection by microscopy. During January 29–March 11, 2020, consecutive patients of varying ages experiencing symptoms of malaria who sought care at Général Peltier Hospital, Djibouti City, were tested by malaria RDT with the First Response Malaria Ag (pLDH/HRP2) Combo Card Test (Premier Medical Corporation, https://www.premiermedcorp.com) and routine venipuncture for hematologic and electrolyte profiling.

### Dried Blood Spot Creation

Dried blood spots (DBS) were prepared from remaining venous blood with 50–75 µL of remnant in EDTA tubes spotted on Whatman Protein Saver cards 903 or Whatmann 3M filter paper (Cytiva Life Sciences, https://www.cytivalifesciences.com). The spots were dried for >4 hours at room temperature and then stored with desiccant in sealable bags and shipped to the CDC (Atlanta, GA, USA).

### *Plasmodium* Antigen Detection by Laboratory Multiplex Assay

DBS processing and testing for *Plasmodium* antigens were performed at CDC as described previously (*14*,*15*). A 6-mm punch was taken from each DBS for elution in blocking buffer (final whole blood dilution of 1:20) for the bead-based multiplex antigen detection assay of pan*-Plasmodium* aldolase and lactate dehydrogenase (LDH), HRP2 (and HRP3), and *P. vivax* LDH (PvLDH). Assay plates were run on a MAGPIX machine (Luminex Corp., https://www.luminexcorp.com) with a target of 50 beads per region.

### *Plasmodium* Species Identification by PCR and *pfhrp2* and *pfhrp3* Genotyping

We selected DBS samples positive for any *Plasmodium* antigens for DNA extraction (DNA Mini Kit; QIAGEN, https://www.qiagen.com) and *Plasmodium* species–specific photo-induced electron transfer (PET) PCR and quantification of DNA (*16*). Samples positive for *P. falciparum* DNA had nested PCR reactions for single-copy *pfmsp1* and *pfmsp2* genes as quality control for DNA quantity and integrity (*17*,*18*). We further assayed only those samples amplifying both control genes to determine presence or absence of *pfhrp2* and *pfhrp3* genes. Genotyping for *pfhrp2* was performed by a single-step PCR amplifying *pfhrp2* inclusive of both exons (*19*), and genotyping for *pfhrp3* was through 2 nested PCR reactions with primers specific for exon 1–2 and exon 2 regions (*17*,*18*). All genotyping reactions were run by 2 independent operators on different days and by a third operator if amplification results were discordant.

### Genetic Haplotypes through Neutral Microsatellites

To assess multiplicity of infection and relatedness of *P. falciparum* parasites, we selected 7 neutral microsatellite (NMS) genetic markers: TA1 on chromosome 6, poly-α on chromosome 4, PfPK2 on chromosome 12, 2490 on chromosome 10, C2M34–313 on chromosome 2, C3M69–383 on chromosome 3, and TA109 on chromosome 6 (*10*,*20*). We determined the sizes of the amplification products by capillary electrophoresis on an Applied Biosystems 3130xl Genetic Analyzer (ThermoFisher Scientific, https://www.thermofisher.com) and analyzed data by using GeneMarker version 3.0.0 (SoftGenetics, https://softgenetics.com). We considered infections monoclonal if all 7 NMS had only 1 allele call. We used NMS data from previous studies to compare Djibouti results to *P. falciparum* parasites from other countries (Appendix Table 1).

### Statistical Analysis

We compared lognormal means for HRP2/3 antigen concentration by genotype by using the 2-tailed Student *t* test with equal variance. For secondary analyses, we divided antigen assay signals for the entire sample set into low and high HRP2/3 antigen levels by comparing them with levels of the pLDH and pAldolase antigens as described previously (*21*). In brief, if blood samples were positive for pan-*Plasmodium* LDH or aldolase and negative for HRP2/3 antigens or had atypically low amounts of HRP2/3 relative to the pan-*Plasmodium* targets, we selected them as high-priority specimens with phenotypic evidence for *pfhrp2/3* gene deletions (Appendix Figure 1).

We assessed NMS data and measures of relatedness by using the PopGenReport package in R (R Foundation for Statistical Computing, https://www.r-project.org) (*22*). For all countries, we used monoclonal infections and polyclonal infections with distinct haplotypes or dominant haplotypes for the relatedness and principal component analysis.

## Results

**Figure 1:**
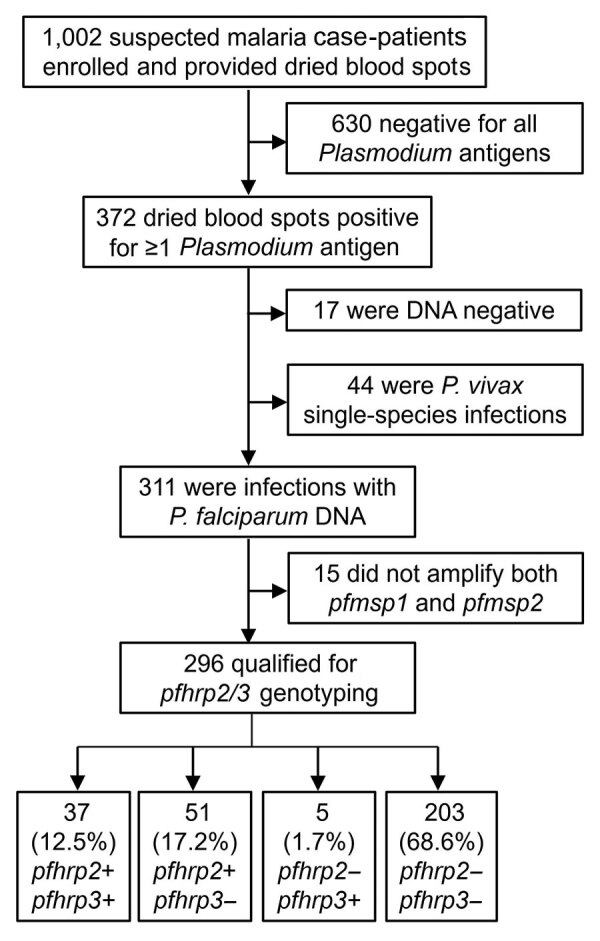
Flow diagram for reporting *pfhrp2* and *pfhrp3* genotype for all specimens in study of *Plasmodium falciparum* parasites with *pfhrp2* and *pfhrp3* deletions, Djibouti, 2019–2020. Terminal boxes display number of samples successfully genotyped for *pfhrp2/3*.

During January 29–March 20, 2020, of the suspected malaria cases registered at Général Peltier Hospital, 998 DBS were collected; an additional 4 samples previously collected in December 2019 were included in the sample set because they were identified as highly suspicious of *pfhrp2/3* deletions on the basis of RDT results (Figure 1). Laboratory antigen screening revealed 630 (62.9%) samples were negative for all antigens; those samples were not investigated further because there was no suspicion of *Plasmodium* spp. infection. 

We extracted DNA from the remaining 372 (37.1%) *Plasmodium* antigen–positive samples for further molecular investigation. Of those 372 samples, detectable *Plasmodium* DNA was absent in 17 (4.6%) samples, and 44 samples (11.8%) were found to be *P. vivax* single-species infections; samples within these 2 groups were not considered further for *pfhrp2/3* deletion reporting. Of the remaining 311 samples containing *P. falciparum* DNA, 15 (4.8%) did not amplify both *pfmsp1* and *pfmsp2* single-copy genes and were not eligible for reporting of *pfhrp2/3* genotypes. Of the 296 *P. falciparum* infections that qualified for reporting genotyping, 37 (12.5%) were a genotype of *pfhrp2+/pfhrp3+*, 51 (17.2%) were *pfhrp2+/pfhrp3*–, 5 (1.7%) were *pfhrp2*–*/pfhrp3+*, and 203 (68.6%) were a double-deletion genotype of *pfhrp2*–*/pfhrp3*– (Figure 1). For all 296 samples of *P. falciparum* infections successfully genotyped, 208 (70.3%) demonstrated a *P. falciparum* infection with *pfhrp2* deletion and 254 (85.8%) showed deletion in the *pfhrp3* gene.

Using the hypothetical selection method (see Methods) based on levels of pan-*Plasmodium* antigens compared with HRP2/3 antigens, of the 372 antigen-positive samples, 241 (64.8%) had a complete absence of HRP2/3 assay signal, 20 (5.4%) had an atypically low amount of HRP2/3 relative to other pan-*Plasmodium* antigens, and 111 (29.8%) had high levels of HRP2/3 relative to other pan-*Plasmodium* antigens (Appendix Figure 1). Of all 241 samples negative for HRP2/3 signal, 183 (75.9%) were appropriate for *pfhrp2/3* genotyping, and all demonstrated a *pfhrp2* deletion. When categorized into the low HRP2/3 category, of the 18 samples that qualified for genotyping, 6 (33.3%) showed a deletion of the *pfhrp2* gene. If categorized into the high HRP2/3 category, of 90 samples that qualified for genotyping, the *pfhrp2* gene was amplified in most (74 [82.2%]).

**Figure 2:**
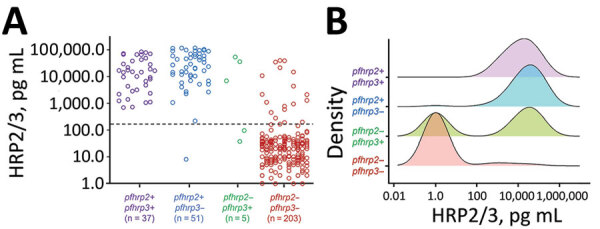
Distributions of HRP2/HRP3 antigen concentrations by *pfhrp2* and *pfhrp3* genotype for specimens in study of *Plasmodium falciparum* parasites with *pfhrp2* and *pfhrp3* deletions, Djibouti, 2019–2020. A) Individual antigen concentrations for all 296 samples successfully genotyped for *pfhrp2/3.* Dashed line denotes the assay level of quantitation. B) Smoothed kernel density plots for log-transformed HRP2/3 concentration by the four *pfhrp2/3* genotypes. HRP2/3, histidine-rich protein 2/3.

We found that concentrations of the HRP2 antigen (with potential supplemented signal from HRP3) were strongly associated with the different *pfhrp2/3* genotypes (Figure 2). With the exception of 1 specimen, all samples found to be positive for the *pfhrp2* gene (n = 88) showed high concentrations of the HRP2/3 antigen; the *pfhrp2+/pfhrp3+* genotype samples showed a log-normal mean concentration of 10,794 pg/mL, and the *pfhrp2+/pfhrp3*– genotype samples showed a log-normal mean concentration of 18,017 pg/mL (Figure 2, panel A). Blood samples from infections with *P. falciparum* parasites lacking the *pfhrp2* gene showed, on average, much lower concentrations of HRP2/3 antigens: log-normal mean of 421 pg/mL in *pfhrp2*–*/pfhrp3+* samples and log-normal mean of 2.0 pg/mL in *pfhrp2*–*/pfhrp3*– samples. Concentration was significantly lower in the blood samples from *pfhrp2*–*/pfhrp3*– parasite infections than in *pfhrp2+/pfhrp3+* (p<0.001), *pfhrp2+/pfhrp3*– (p<0.001), and *pfhrp2*–*/pfhrp3+* (p = 0.049) parasite infections. Of the 5 infections with *pfhrp2*–*/pfhrp3+* parasites, 2 (40.0%) showed an absence of HRP2/3 antigen signal, compared with 185/203 (91.1%) of *pfhrp2*–*/pfhrp3*– infections. Density plots of HRP2/3 antigen concentration by infecting parasite genotype illustrate these trends by genotype category (Figure 2, panel B).

**Figure 3:**
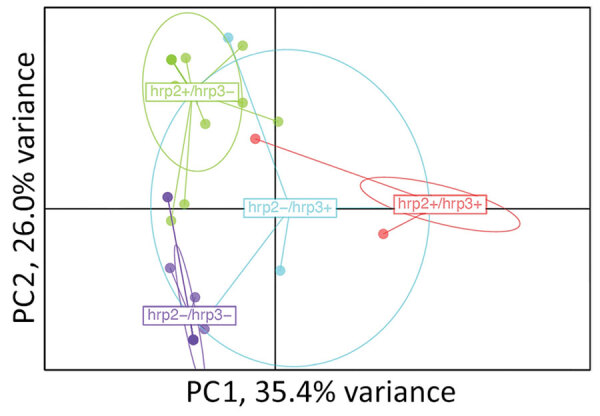
Relatedness of *Plasmodium falciparum* parasites from Djibouti, 2019–2020, with different *pfhrp2* and *pfhrp3* genotypes. Cluster PC analysis shown for 7 neutral microsatellite data for monogenomic infections by subpopulations: *pfhrp2+/pfhrp3+* (n = 16), *pfhrp2+/pfhrp3*– (n = 15), *pfhrp2*–*/pfhrp3+* (n = 4), *pfhrp2*–*/pfhrp3*– (n = 17). Plot shown with PC1 on x-axis and PC2 on y-axis with 95% confidence ellipses. PC, principal component.

In total, 65 of the Djibouti infections were chosen for NMS sequencing: 20 *pfhrp2+/pfhrp3+*, 20 *pfhrp2+/pfhrp3*–, 5 *pfhrp2*–*/pfhrp3+*, and 20 *pfhrp2*–*/pfhrp3*–. Of those, 52/65 (80.0%) were found to be monoclonal infections, and this finding did not differ significantly by genotype: *pfhrp2+/pfhrp3+* (16/20 [80.0%]), *pfhrp2+/pfhrp3*– (15/20 [75.0%]), *pfhrp2*–*/pfhrp3+* (4/5 [80.0%]), *pfhrp2*–*/pfhrp3*– (17/20 [85.5%]). Three of the genotypes (all except for *pfhrp2*–*/pfhrp3+*) showed a high degree of independent clustering by principal component analysis (Figure 3). Both the Jost pairwise D and Hendricks pairwise G_ST_ measures of gene differentiation found the *pfhrp2*–*/pfhrp3*– genotype to be most related to *pfhrp2*–*/pfhrp3+* parasites (Appendix Table 2).

**Figure 4:**
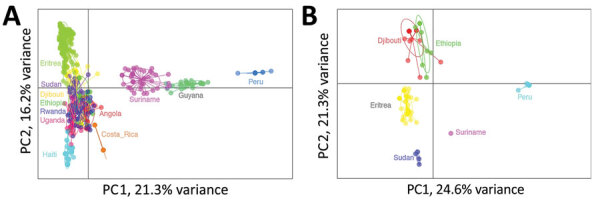
Relatedness of *Plasmodium falciparum* parasites from Djibouti, 2019–2020 with other global isolates. A) Cluster PC analysis shown for neutral microsatellite data for monogenomic infections by collection from different countries: Angola (n = 32), Costa Rica (n = 14), Djibouti (n = 52), Eritrea (n = 187), Ethiopia (n = 20), Guyana (n = 27), Haiti (n = 86), Peru (n = 18), Rwanda (n = 42), Sudan (n = 37), Suriname (n = 44), Uganda (n = 25). B) Cluster PC analysis shown for neutral microsatellite data for monogenomic infections containing *pfhrp2* deletions by collection from different countries: Djibouti (n = 21), Eritrea (n = 43), Peru (n = 18), Ethiopia (n = 8), Sudan (n = 4), and Suriname (n = 1). Plots shown with PC1 on x-axis and PC2 on y-axis and 95% confidence ellipses. PC, principal component.

Comparing relatedness of *P. falciparum* by country (regardless of *pfhrp2/3* genotype) found distinct clustering by the area of the world in which the parasites originated (Figure 4, panel A). Both Jost pairwise D and Hendricks pairwise G_ST_ found parasites from Djibouti and other parts of Africa more related to each other than to isolates from South America or Asia (Appendix Table 3, Figure 2). When repeating the analysis with only parasites from Africa, Djibouti and Ethiopia parasites were found to be most related in comparison with *P. falciparum* from the other 5 African countries (Appendix Figure 3). For global isolates with *pfhrp2* deletions, South America and Africa parasites were not strongly related to each other; Peru and Suriname parasites had the highest principal component 1 values as well as highest Jost pairwise D and Hendricks pairwise G_ST_ in comparison with African *pfhrp2*-deleted parasites (Table; Figure 4, panel B). The *pfhrp2* deleted parasites from Sudan, Eritrea, Ethiopia, and Djibouti showed relatively similar principal component 1 values, and Ethiopia and Djibouti *pfhrp2*-deleted genotypes demonstrated the closest overall clustering. Among these deleted parasites, Djibouti *P. falciparum* was most closely related to Ethiopia (pairwise G_ST_ 0.68) *pfhrp2*-deleted *P. falciparum*, followed by Eritrea (0.77), Sudan (0.97), Peru (0.98), and Suriname (0.99)*.* If assessing relatedness among parasites lacking only the *pfhrp3* gene, we noted similar findings; the highest degree of relatedness was seen among the Djibouti and Ethiopia parasites (Appendix Figure 4).

**Table:** Genetic relatedness of *pfhrp2*-deleted *Plasmodium falciparum* parasites from Djibouti, 2019–2020, compared with those from other countries*

Comparison	Country	Djibouti	Eritrea	Ethiopia	Peru	Sudan
Jost D pairwise	Djibouti					
	Eritrea	0.530				
	Ethiopia	0.444	0.704			
	Peru	0.873	0.888	0.846		
	Sudan	0.920	0.817	0.879	0.994	
	Suriname	0.987	0.831	1.00	0.795	0.962
Hendrick pairwise G_ST_	Djibouti					
	Eritrea	0.772				
	Ethiopia	0.679	0.843			
	Peru	0.977	0.972	0.953		
	Sudan	0.972	0.917	0.941	0.999	
	Suriname	0.998	0.949	1.000	0.991	0.990

*Darker shading indicates higher level of relatedness.

## Discussion

Confirmatory diagnosis of *P. falciparum* malaria through testing for the presence of HRP2 antigen by RDT has been a substantial improvement for providing appropriate case management in many malaria-endemic countries. Discovery of *pfhrp2* gene deletions in natural *P. falciparum* populations has led to doubts about the sustained use of this antigen for diagnostic purposes (*1*). To date, most *P. falciparum*–endemic settings that rely heavily on RDTs for routine diagnostics have been found to have populations of parasites that express high amounts of HRP2 and HRP3 antigens (*8*,*15*,*18*,*21*,*23*,*24*). The ability to evade HRP2-based diagnostics (and subsequent treatment) might lead to a selective advantage for parasites with gene deletions (*25*). Routine surveillance of *P. falciparum*–endemic populations is required to ensure that primary diagnostic tools are still accurate.

Multiple recent studies in the Horn of Africa have demonstrated a high proportion of *P. falciparum* in the region with *pfhrp2* gene deletions and that most isolates also have *pfhrp3* deletions. A 2016 Eritrea health facility survey found 62.0% of *P. falciparum* infections consisted of parasites with both *pfhrp2* and *pfhrp3* deletions, all of which were also HRP2-negative by RDT (*10*). Of persons enrolled in health facility surveys during 2017–2018 in northern Ethiopia, *pfhrp2* deletions were responsible for between 11.5%–14.9% of false-negative HRP2-RDT results (*12*). A survey of 3 health facilities in Djibouti in early 2019 also found these concerning results: in a small sample set of 79 *P. falciparum* PCR-positive patients, 86.5% demonstrated *pfhrp2/3* deletions (*13*).

In light of previous findings, this 2019–2020 study was designed to assess the prevalence of *pfhrp2/3* deletions in Djibouti City, relatedness of Djibouti *P. falciparum* to other global isolates, and overall relatedness of *P. falciparum* within the Horn of Africa compared with other global sites. Most (63%) of the 1,002 DBS samples collected from persons with malaria-like symptoms were negative for any *Plasmodium* antigens and did not undergo molecular assays because *Plasmodium* infection was not suspected. The bead-based antigen assay has been shown to detect *Plasmodium* infection at approximately the same level as standard PCR assays at ≈2 parasites/uL blood (*15*). Among the remaining 372 *Plasmodium*-positive samples, only 111 (29.8%) displayed moderate to high levels of HRP2/3 antigen profile indicative of *P. falciparum* with functional *pfhrp2* or *pfhrp3* genes. By those *Plasmodium* antigen data alone, the pervasiveness of low or absent HRP2 levels in 70% of samples from symptomatic *Plasmodium*-infected persons raised suspicion of either a high frequency of non–*P. falciparum* infections or high prevalence of *P. falciparum* isolates with the *pfhrp2/3* genes deleted; both scenarios were found to be true in Djibouti. Of the 355 samples with detectable *Plasmodium* DNA by PET-PCR, 44 (12.4%) were single-species *P. vivax* infections; this finding is in line with previous reports of *P. vivax* in Djibouti, as well as in neighboring Ethiopia and Eritrea (*9*,*26*). Most (95%) of the 311 samples with *P. falciparum* DNA were successfully genotyped for single-copy control genes (*17*), reflecting high-density infections in the symptomatic study population. Of the 296 *P. falciparum* infections with reportable genotyping results, only 37 (12.5%) contained wild-type parasites with both *pfhrp2* and *pfhrp3* genes amplified. Only *pfhrp3* was deleted in 17.2% of parasites, only *pfhrp2* was deleted in 1.7%, and more than two thirds of all infections (68.6%) were from *P. falciparum* lacking both genes. The 68.6% prevalence of double-deleted parasites in Djibouti City is lower than the 86.5% previously reported by Iriart et al. (*13*). This high prevalence of *pfhrp2/3* dual-deleted infections, coupled with our finding that most infections are monoclonal (80%), suggests that a high percentage of *P. falciparum* infections in Djibouti City would not be detected by HRP2-based RDTs. This level is well beyond the 5% threshold recommended by the WHO to consider a replacement of exclusive HRP2-based diagnostics for detecting *P. falciparum* (*27*), and the findings from our study have already been shared with the Djibouti Ministry of Health and WHO regional partners.

Regarding relatedness to other global isolates, the parasites found in Djibouti (regardless of genotype) clustered closely with *P. falciparum* haplotypes from Africa and showed greater distance to *P. falciparum* from the New World and Asia. Djibouti City is a large port city located on the east central coast and is home to approximately half the country’s population. Because Djibouti City is a large center of trade and population movement, some enrolled patients might have contracted *P. falciparum* infection in a country other than Djibouti, but travel history for participants was unavailable for this study. However, the objective genetic data show that even if some infections were acquired outside Djibouti, they all appear to be Africa-derived from more proximal countries. High relatedness (low diversity) of *P. falciparum* within Djibouti has been previously observed for isolates collected throughout the country within individual surveys and without substantial differences among multiple years of collection (*28*), although genotyping for *pfhrp2/3* deletions was not performed. The relatedness of Djibouti and Ethiopia *pfhrp2*-deleted parasites observed in this study was closer when compared with Eritrea or Sudan isolates and very distant from *pfhrp2*-deleted *P. falciparum* from Peru and Suriname. The same finding was noted for *pfhrp3*-only deleted parasites, where Djibouti and Ethiopia populations practically overlie each other. This evidence points to de novo gene deletions and expansion of these deleted *P. falciparum* populations in the Horn of Africa rather than importation of deleted parasites from other areas of the world. Specifically for the Horn of Africa, close background lineages by NMS data for *pfhrp2*- and *pfhrp3*-deleted parasites from Djibouti and Ethiopia points to an expansion of common gene-deleted populations that exist in these adjacent countries. Eritrea parasite lineages from both wild-type and deleted *P. falciparum* appear to be differentiated from the Ethiopia/Djibouti lines, suggesting separate *pfhrp2/3* deletion events on unique *P. falciparum* strains and a more distant common ancestor.

This study and its findings are subject to limitations. Though many patients exhibiting malaria symptoms were enrolled in Djibouti City, no other areas of the country are represented by this sample set, so these conclusions and estimates could not necessarily be applied nationwide. However, Djibouti City accounts for 95% of the country’s malaria case load and Général Peltier Hospital is the largest hospital in the region, and previous reports have found *P. falciparum* in Djibouti to be of overall low diversity (*28*,*29*). Furthermore, the Djibouti Ministry of Health considered these results sufficiently representative to mandate a nationwide RDT policy change. Without the recent travel history of enrolled participants, we cannot state all *P. falciparum* parasites analyzed in this study originated in Djibouti. Quality microscopy could not be performed uniformly on these blood samples, so we were unable to obtain visual confirmation of *P. falciparum*. In addition, because of laboratory workflow, RDT results from enrollment could not be reliably linked with venous blood samples and therefore were not compared directly with laboratory data. However, the 4 samples collected in December 2019 that triggered this investigation demonstrated *pfhrp2/3* deletions causing known false-negative results by HRP2-based RDT.

In conclusion, results from both antigen detection and *pfhrp2/3* molecular genotyping provide evidence of a high prevalence of symptomatic malaria cases in Djibouti caused by *P. falciparum* lacking functional *pfhrp2/3* genes. These findings, coupled with high occurrence of monoclonal infections and single *pfhrp2*-deleted infections, suggest that nearly 70% of HRP2-based RDTs would return negative results for *P. falciparum* infection in Djibouti, which is expected to have serious negative health impacts on the community. Djibouti *P. falciparum* parasites with gene deletions are most closely related to other parasites in the Horn of Africa with a recent common ancestor or routine importation from Ethiopia. Gene-deleted haplotypes show no evidence of importation from South America.

Appendix Additional information about *Plasmodium falciparum* *pfhrp2* and *pfhrp3* gene deletions and relatedness to other global isolates, Djibouti, 2019–2020
