# Supplementary material for: Plasmodium falciparum pfhrp2 and pfhrp3 Gene Deletions and Relatedness to Other Global Isolates, Djibouti, 2019–2020
Source: Emerg Infect Dis. 2022 Oct;28(10):2043–50. doi: 10.3201/eid2810.220695 (PMC9514350; doi:10.3201/eid2810.220695)
Supplement: Appendix — Additional information about Plasmodium falciparum pfhrp2 and pfhrp3 gene deletions and relatedness to other global isolates, Djibouti, 2019–2020 [file 22-0695-Techapp-s1.pdf]

Article DOI: https://doi.org/10.3201/eid2810.220695

Plasmodium falciparum pfhrp2 and *pfhrp3* Gene Deletions and Relatedness to Other Global Isolates, Djibouti, 2019–2020

Appendix

Appendix Table 1. Source of NMS data from previous studies

Country	Year(s)	Samples with NMS data used in current study (n)	Reference
Angola	2019	32	(*1*)
Costa Rica	2019	14	(*2*)
Eritrea	2019	173	(*3*)
Eritrea	2016	14	(*4*)
Ethiopia	2018	20	(*5*)
Guyana	2010	27	(*6*)
Haiti	2016	86	(*7*)
Indian Subcontinent†	2013–2016	11	(*8*)
Malaysia*	2008–2014	27	(*9*)
Peru	2010–2012	18	(*10*)
Rwanda	2018	42	(*11*)
Sudan	2010–2018	37	(*12*)
Suriname	2013–2014	43	(*13*)
Uganda	2018	25	(*14*)

*NMS, neutral microsatellite.
†Inclusive of India and Pakistan. Did not include complete 7 NMS panel, so used only in supplementary analysis.

Appendix Table 2. Jost's D and Hendrick Gst relatedness of Djibouti *P. falciparum* by *pfhrp2* and *pfhrp3* genotype*

Comparison	Genotypes	pfhrp2+/pfhrp3+	pfhrp2+/pfhrp3-	pfhrp2-/pfhrp3+	pfhrp2-/pfhrp3-
Jost's D Pairwise	pfhrp2+/pfhrp3+				
	pfhrp2+/pfhrp3-	0.734			
	pfhrp2-/pfhrp3+	0.395	0.475		
	pfhrp2-/pfhrp3-	0.721	0.506	0.346	
Hendrick Pairwise Gst	pfhrp2+/pfhrp3+				
	pfhrp2+/pfhrp3-	0.924			
	pfhrp2-/pfhrp3+	0.662	0.691		
	pfhrp2-/pfhrp3-	0.948	0.832	0.630	

*Darker shading indicates higher level of relatedness.

Appendix Table 3. Jost's D and Hendrick Gst relatedness of Djibouti *Plasmodium falciparum* compared to *P falciparum* from other countries*

Comparison	Genotypes	Djibouti	Angola	Costa Rica	Eritrea	Ethiopia	Guyana	Haiti	Peru	Rwanda	Sudan	Suriname
Jost's D Pairwise	Djibouti											
	Angola	0.699										
	Costa Rica	0.924	0.746									
	Eritrea	0.636	0.786	0.977								
	Ethiopia	0.486	0.670	0.894	0.752							
	Guyana	0.949	0.875	0.895	0.985	0.954						
	Haiti	0.657	0.630	0.821	0.882	0.634	0.999					
	Peru	0.926	0.864	0.895	0.970	0.903	0.460	1.00				
	Rwanda	0.586	0.478	0.875	0.724	0.516	0.932	0.519	0.966			
	Sudan	0.772	0.600	0.943	0.756	0.744	0.966	0.731	0.997	0.603		
	Suriname	0.867	0.919	0.917	0.813	0.916	0.514	0.925	0.804	0.905	0.892	
	Uganda	0.598	0.496	0.924	0.791	0.494	0.970	0.474	0.993	0.375	0.633	0.941
Hendrick Pairwise Gst	Djibouti											
	Angola	0.796										
	Costa Rica	0.965	0.844									
	Eritrea	0.761	0.861	0.990								
	Ethiopia	0.610	0.758	0.944	0.835							
	Guyana	0.976	0.933	0.957	0.993	0.976						
	Haiti	0.803	0.769	0.921	0.941	0.769	1.00					
	Peru	0.976	0.948	0.976	0.991	0.963	0.802	1.00				
	Rwanda	0.702	0.587	0.928	0.816	0.620	0.964	0.676	0.988			
	Sudan	0.849	0.699	0.969	0.838	0.816	0.982	0.839	0.999	0.700		
	Suriname	0.927	0.953	0.961	0.895	0.950	0.714	0.966	0.938	0.944	0.936	
	Uganda	0.716	0.609	0.958	0.866	0.604	0.985	0.641	0.998	0.486	0.730	0.966

*Darker shading indicates higher level of relatedness.

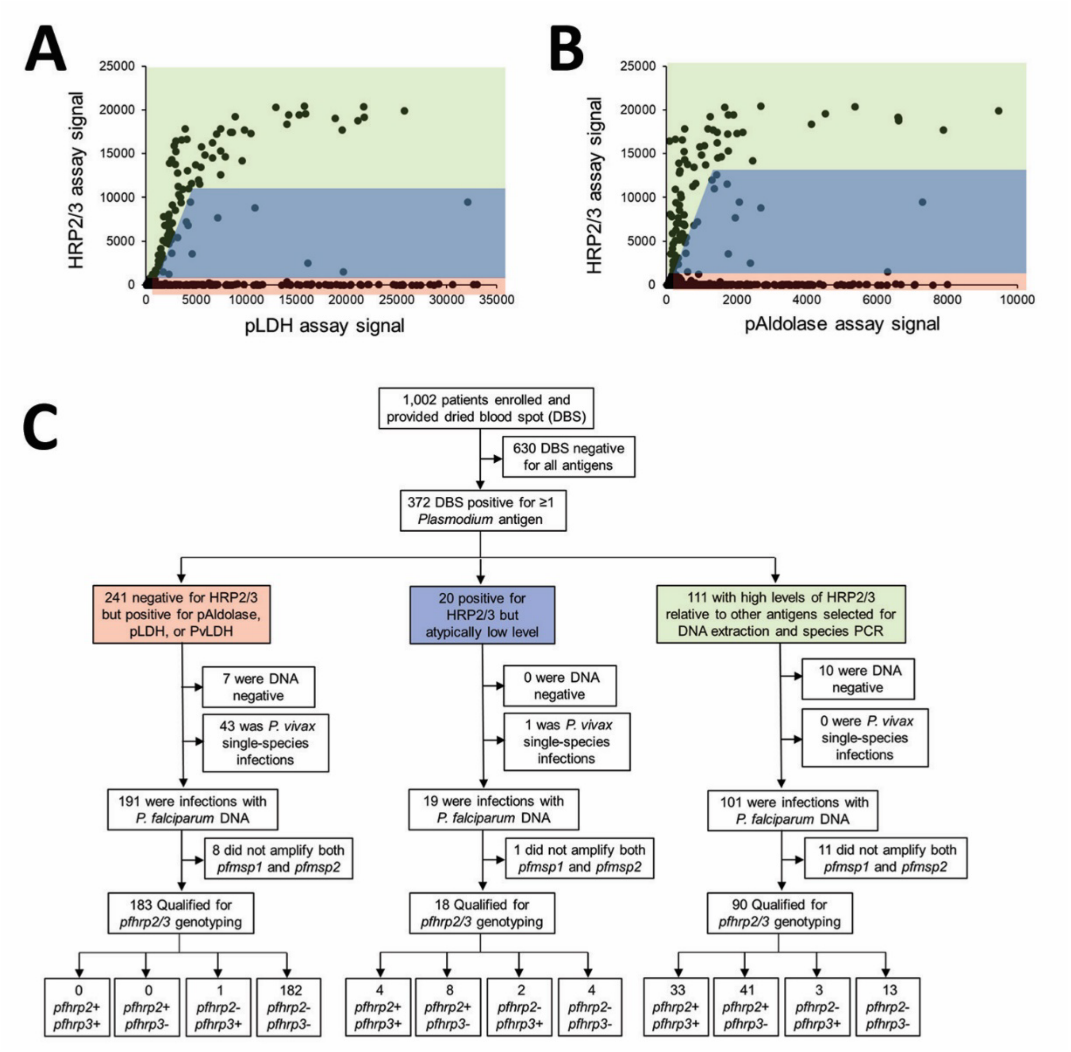

Appendix Figure 1. Comparison of the HRP2/3 assay signal with other pan-*Plasmodium* antigen targets to categorize sample set into high and low HRP2/3 levels. Scatterplots for HRP2/3 immunoassay signal as compared to pan-*Plasmodium* lactate dehydrogenase (A) or pan-*Plasmodium* aldolase (B) signals. C) Flowchart for genotype reporting for all samples if dichotomizing by HRP2/3 category. For all panels, red shading indicates complete absence of HRP2/3 assay signal, blue shading indicates relatively low HRP2/3 signal when compared with pan-*Plasmodium* targets, and green shading indicates high HRP2/3 signal compared with pan-*Plasmodium* targets.

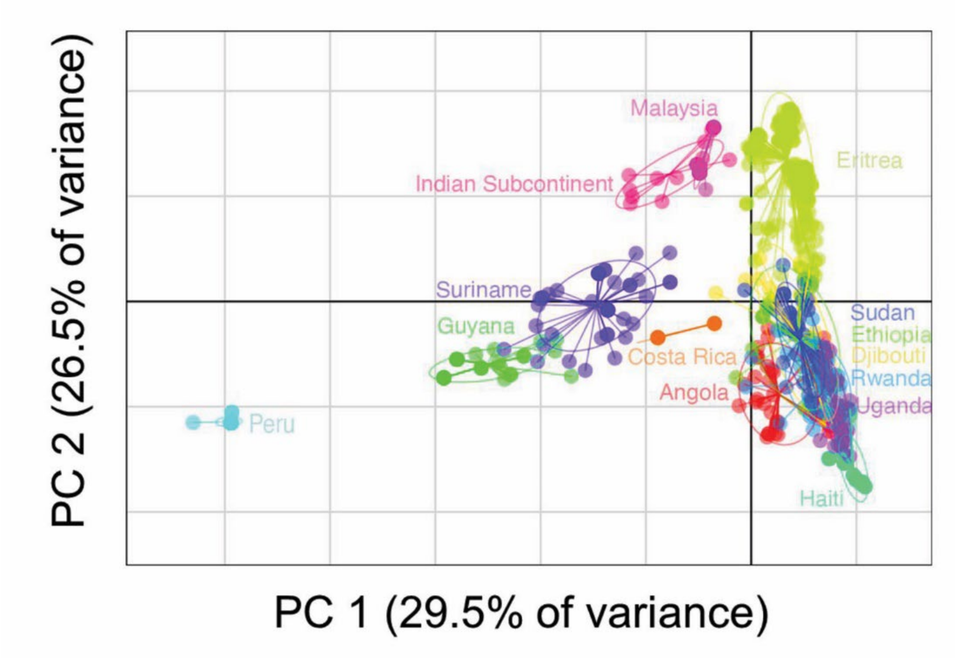

Appendix Figure 2. Relatedness of Djibouti *Plasmodium falciparum* parasites with other global isolates with limited neutral microsatellite data. Inclusion of Asian isolates with only 4 neutral microsatellite targets published (TA1, Polyα, PfPK2, 2490). Plots shown with PC1 on x-axis and PC2 on y-axis, with 95% confidence ellipses

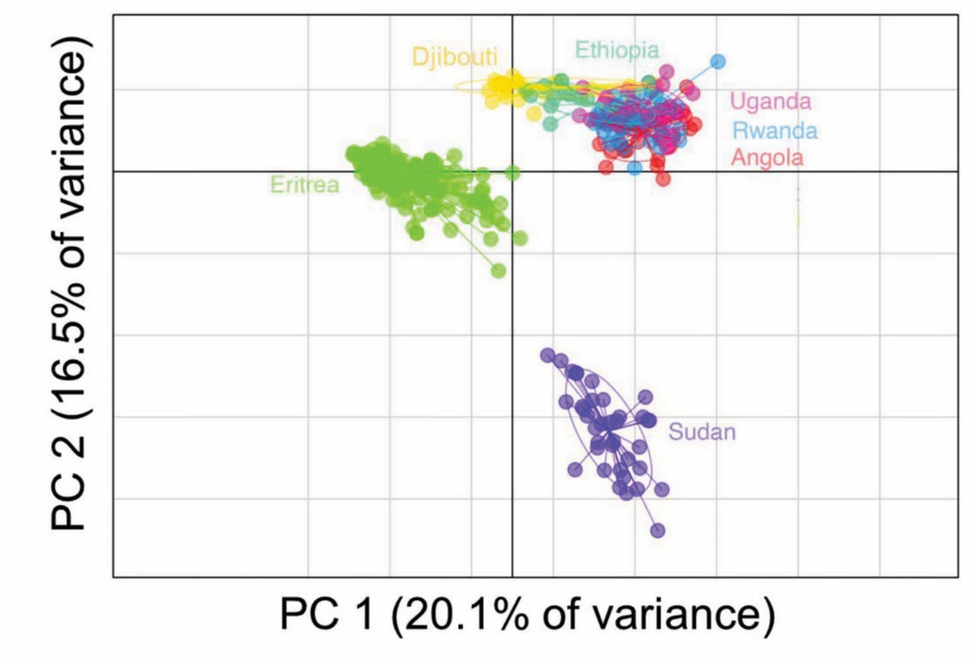

Appendix Figure 3. Relatedness of Djibouti *Plasmodium falciparum* parasites with other African isolates. Plots shown with PC1 on x-axis and PC2 on y-axis, with 95% confidence ellipses.

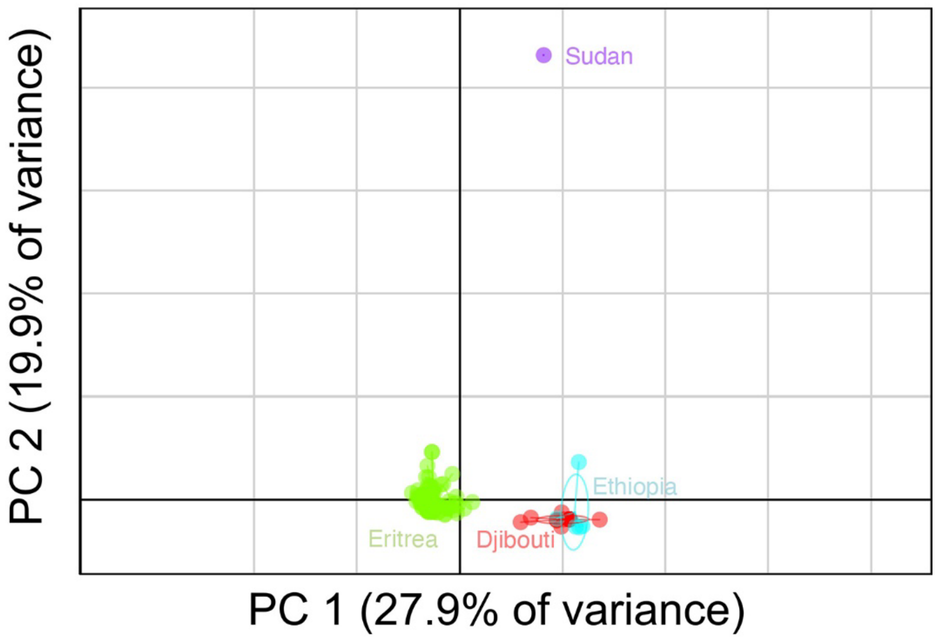

Appendix Figure 4. Relatedness of Djibouti *Plasmodium falciparum* parasites with other African isolates lacking only *pfhrp3*. Plots shown with PC1 on x-axis and PC2 on y-axis, with 95% confidence ellipses.

References

1. Dimbu PR, Horth R, Cândido ALM, Ferreira CM, Caquece F, Garcia LEA, et al. Continued low efficacy of artemether-lumefantrine in Angola in 2019. Antimicrob Agents Chemother. 2021;65:e01949-20. PubMed https://doi.org/10.1128/AAC.01949-20

2. Santamaría AM, Vásquez V, Rigg C, Moreno D, Romero L, Justo C, et al. *Plasmodium falciparum* genetic diversity in Panamá based on *glurp*, *msp*-1 and *msp*-2 genes: implications for malaria elimination in Mesoamerica. Life (Basel). 2020;10:E319. PubMed https://doi.org/10.3390/life10120319

3. Mihreteab S, Anderson K, Pasay C, Smith D, Gatton ML, Cunningham J, et al. Epidemiology of mutant *Plasmodium falciparum* parasites lacking histidine-rich protein 2/3 genes in Eritrea 2 years after switching from HRP2-based RDTs. Sci Rep. 2021;11:21082. PubMed https://doi.org/10.1038/s41598-021-00714-8

4. Berhane A, Anderson K, Mihreteab S, Gresty K, Rogier E, Mohamed S, et al. Major threat to malaria control programs by *Plasmodium falciparum* lacking histidine-rich protein 2, Eritrea. Emerg Infect Dis. 2018;24:462–70. PubMed https://doi.org/10.3201/eid2403.171723

5. Leonard CM, Mohammed H, Tadesse M, McCaffery JN, Nace D, Halsey ES, et al. Missed *Plasmodium falciparum* and *Plasmodium vivax* mixed infections in Ethiopia threaten malaria elimination. Am J Trop Med Hyg. 2021;106:667–70. PubMed https://doi.org/10.4269/ajtmh.21-0796

6. Akinyi Okoth S, Abdallah JF, Ceron N, Adhin MR, Chandrabose J, Krishnalall K, et al. Variation in *Plasmodium falciparum* histidine-rich protein 2 (pfhrp2) and *Plasmodium falciparum* histidine-rich protein 3 (pfhrp3) gene deletions in Guyana and Suriname. PLoS One. 2015;10:e0126805. PubMed https://doi.org/10.1371/journal.pone.0126805

7. Daniels RF, Chenet S, Rogier E, Lucchi N, Herman C, Pierre B, et al. Genetic analysis reveals unique characteristics of *Plasmodium falciparum* parasite populations in Haiti. Malar J. 2020;19:379. PubMed https://doi.org/10.1186/s12936-020-03439-7

8. Al-Rumhi A, Al-Hashami Z, Al-Hamidhi S, Gadalla A, Naeem R, Ranford-Cartwright L, et al. Influx of diverse, drug resistant and transmissible *Plasmodium falciparum* into a malaria-free setting in Qatar. BMC Infect Dis. 2020;20:413. PubMed https://doi.org/10.1186/s12879-020-05111-6

9. Mohd Abd Razak MR, Sastu UR, Norahmad NA, Abdul-Karim A, Muhammad A, Muniandy PK, et al. Genetic diversity of *Plasmodium falciparum* populations in malaria declining areas of Sabah, East Malaysia. PLoS One. 2016;11:e0152415. PubMed https://doi.org/10.1371/journal.pone.0152415

10. Baldeviano GC, Okoth SA, Arrospide N, Gonzalez RV, Sánchez JF, Macedo S, et al. Molecular epidemiology of *Plasmodium falciparum* malaria outbreak, Tumbes, Peru, 2010-2012. Emerg Infect Dis. 2015;21:797–803. PubMed https://doi.org/10.3201/eid2105.141427

11. Uwimana A, Umulisa N, Venkatesan M, Svigel SS, Zhou Z, Munyaneza T, et al. Association of Plasmodium falciparum kelch13 R561H genotypes with delayed parasite clearance in Rwanda: an open-label, single-arm, multicentre, therapeutic efficacy study. Lancet Infect Dis. 2021;21:1120–8. PubMed https://doi.org/10.1016/S1473-3099(21)00142-0

12. Prosser C, Gresty K, Ellis J, Meyer W, Anderson K, Lee R, et al. *Plasmodium falciparum* histidine-rich protein 2 and 3 gene deletions in strains from Nigeria, Sudan, and South Sudan. Emerg Infect Dis. 2021;27:471–9. PubMed https://doi.org/10.3201/eid2702.191410

13. Chenet SM, Okoth SA, Kelley J, Lucchi N, Huber CS, Vreden S, et al. Molecular profile of malaria drug resistance markers of *Plasmodium falciparum* in Suriname. Antimicrob Agents Chemother. 2017;61:e02655-16. PubMed https://doi.org/10.1128/AAC.02655-16

14. Ebong C, Sserwanga A, Namuganga JF, Kapisi J, Mpimbaza A, Gonahasa S, et al. Efficacy and safety of artemether-lumefantrine and dihydroartemisinin-piperaquine for the treatment of uncomplicated *Plasmodium falciparum* malaria and prevalence of molecular markers associated with artemisinin and partner drug resistance in Uganda. Malar J. 2021;20:484. PubMed https://doi.org/10.1186/s12936-021-04021-5
